# A statistical toolbox for metagenomics: assessing functional diversity in microbial communities

**DOI:** 10.1186/1471-2105-9-34

**Published:** 2008-01-23

**Authors:** Patrick D Schloss, Jo Handelsman

**Affiliations:** 1 Department of Microbiology, University of Massachusetts – Amherst, Amherst, MA 01003, USA; 2 Department of Bacteriology, University of Wisconsin – Madison, Madison, WI 53706, USA

## Abstract

**Background:**

The 99% of bacteria in the environment that are recalcitrant to culturing have spurred the development of metagenomics, a culture-independent approach to sample and characterize microbial genomes. Massive datasets of metagenomic sequences have been accumulated, but analysis of these sequences has focused primarily on the descriptive comparison of the relative abundance of proteins that belong to specific functional categories. More robust statistical methods are needed to make inferences from metagenomic data. In this study, we developed and applied a suite of tools to describe and compare the richness, membership, and structure of microbial communities using peptide fragment sequences extracted from metagenomic sequence data.

**Results:**

Application of these tools to acid mine drainage, soil, and whale fall metagenomic sequence collections revealed groups of peptide fragments with a relatively high abundance and no known function. When combined with analysis of 16S rRNA gene fragments from the same communities these tools enabled us to demonstrate that although there was no overlap in the types of 16S rRNA gene sequence observed, there was a core collection of operational protein families that was shared among the three environments.

**Conclusion:**

The results of comparisons between the three habitats were surprising considering the relatively low overlap of membership and the distinctively different characteristics of the three habitats. These tools will facilitate the use of metagenomics to pursue statistically sound genome-based ecological analyses.

## Background

Metagenomics, the culture-independent isolation and characterization of DNA from uncultured microorganisms [1], has facilitated the analysis of the functional biodiversity harbored in the large reservoir of uncultured bacteria and archaea [2-4]. Although early metagenomic studies identified individual genes or activities of interest, recent advances in genome sequencing technologies have made obtaining a complete metagenomic sequence more tractable. Sequence-based approaches combined with functional expression approaches have the potential to identify novel genes important for industrial and ecological applications. Sequence-based approaches have recently been applied to DNA obtained from viruses [5,6], seawater [7-10], wastewater [11,12], sediment [13], sponges [14], acid mine drainage [15], marine worms [16], human gut [17], soil [18], and decomposing whale carcasses [18]. The analysis used to describe these communities has primarily focused on the descriptive characterization and comparison of the relative abundance of proteins that belong to specific functional categories.

Attempts to analyze metagenomic sequences have proven that a metagenomic sequence is more than just a large genome sequencing project. First, the goal of most genome sequence projects is a closed genome sequence where every nucleotide is represented by a desired number of independent sequence reads. In metagenomics, the probability of finding overlapping sequence reads is low in most environments [19]. The probability that overlapping sequence reads are from the same population of bacteria or archaea is even lower so that contigs that are formed are out of necessity chimeras of different genomes that may not even be from the same phylum [20]. Second, a closed genome represents a statistical population of the genes harbored by that organism; therefore, comparing genome sequences for the presence or absence of genes is straightforward. Since it is not possible to close a metagenome, every metagenomic sequence collection represents a statistical sample of the genomes in an environment. Therefore, it is necessary to treat the comparison of communities as a statistical problem. Third, although lab-based cultures that are sequenced do evolve, the differences between lab stocks is minimal compared to the changes faced by natural communities over short periods of time. This makes it difficult to reanalyze a community once a genome sequence has been obtained to improve annotations and understand gene expression.

Five general approaches have been taken to bring statistical analysis to the analysis of metagenomic sequences. The first adapts genomics-based approaches to metagenomics by constructing and curating databases to aid in the annotation and analysis of genes and the contigs they reside on [21,22]. Unfortunately, although such databases provide a critical infrastructure, given the large number of ORFs that have no known function (e.g. 69% in the Sargasso Sea [7]) and the paucity of contigs formed from many sequencing projects (e.g. <1% in the soil [18]), such database searches will be of limited value for comparative metagenomics. The second approach to analyzing metagenomic sequences has been based on the comparison on the relative abundance of annotation categories within the different sequence collections and within databases of assembled genomes [9,10,17,18,23]; these methods implicitly assume that the metagenomic sequences represent a statistical population and/or that the reference databases represent the normal distribution of genes in communities. A third set of approaches attempts to assign a phylogenetic origin for a sequence fragment in the absence of a phylogenetic anchor (e.g. 16S rRNA gene) using nucleotide frequency analysis or sequence signatures [24-27]. Such methods are limited for use with most environments because of the difficulty in forming contigs that are long enough to carry out a robust analysis and assume that the contigs that form are not chimeric. A fourth approach has attempted to compare communities without an annotation. These have attempted to quantify the species richness of communities based on the distribution of sequence read depth among contigs [7] and to compare the diversity of communities based on the relative frequency of different length oligonucleotides in the DNA sequence pool [28]. Finally, there have been attempts to using traditional population biology by analyzing the diversity of specific families of genes found in metagenomic collections [29].

Based on previous metagenomic sequencing efforts, we were interested in developing statistical tools to compare the richness, membership, and structure of the complement of ORFs from multiple communities in which assembly of the entire genomes is not possible. To address this problem, we adapted a set of statistical tools designed to analyze collections of 16S rRNA gene sequences to the analysis of protein coding genes [30-33]. Our goal was to provide additional tools to make statistical and ecological inferences using metagenomic sequence data. Instead of using a traditional pairwise DNA distance matrix obtained from a sequence alignment of homologous genes as is done with 16S rRNA genes, we used BLAST score ratios (BSRs) to develop a distance matrix that represents the similarity of ORFs across homologous groups [34]. To make comparisons among communities, we propose grouping ORFs into operational protein families (OPFs) which are analogous to operational taxonomic units (OTUs) derived from 16S rRNA gene sequences.

## Results and discussion

### A new distance matrix

The goal of this aspect of the work was to develop a method to compare sequence alignments that circumvented the considerable computational effort required to obtain every possible global sequence alignment and pairwise distance. We used local alignments provided in BLAST and the resulting pairwise BLAST scores to generate BSRs. The BSRs approximate the fraction of identical amino acids between two peptide fragments so that a BSR value of 0.30 between two fragments means that they are approximately 30% identical over their full length. By analogy to the analysis of 16S rRNA gene sequences of uncultured bacteria where OTUs are developed based on a distance matrix, we propose using BSR values to define OPFs. Depending on the goals of the analysis an OPF can be defined as necessary. For illustrative purposes and based on previous implementations of BSRs for comparative genomics applications [34,35], unless otherwise indicated we will operationally define an OPF as a collection of fragments that have a BSR greater than 0.30.

To assess the feasibility of using peptide fragments from individual sequence reads, we identified peptide fragments from the individual sequence reads used to assemble the *Bacillus anthracis*, str. Ames genome (GenBank Accession NC_003997, [36]), which contains 4,514 ORFs that were longer than 100 aa. From the individual sequence reads, we identified 92,220 peptide fragments longer than 100 aa. The computational effort required for the pairwise alignment and distance calculation among 92,220 ORFs was prohibitive. Because we expected a majority of the peptide pairs would not have significant similarity, we used BLAST to identify those comparisons that had significant similarity and to calculate BSRs as a surrogate for similarity or distance values (distance = 1-BSR). Instead of generating a 92,220 × 92,220 matrix with 8.5 × 10^9 ^values, we took advantage of the sparseness of the matrix to simplify the calculations and construct a set of three linked-lists in which each list contained the row, column, and BSR values of the full BSR matrix. Since the BSR for a peptide fragment compared to itself is 1.0 and the BSR for a non-significant comparison is 0.0, the corresponding entries in the linked lists could be removed. Once this was completed, there were 2.1 × 10^6 ^values, which represented a significant reduction in the memory required to store the data.

### MG-DOTUR

To assign peptide fragments to OPFs we rewrote the computer code for DOTUR to be compatible with sparse BSR matrices. DOTUR is used to assign collections of 16S rRNA gene sequences and to use the resulting frequency distribution of sequences among OTUs to estimate richness and diversity (Table 1). By analogy, MG-DOTUR assigns peptide fragments to OPFs and estimates the richness and diversity of OPFs for any desired OPF definition. Two classes of methods are available to estimate richness based on frequency distributions. The first uses parametric distributions such as the lognormal distribution to predict the number of unseen groups in a community [37]. Although it is often assumed that microbial communities follow a lognormal distribution, there are no published examples in the microbial ecology literature for which the observed data support such an assumption. This is primarily due to the difficulty in obtaining a sufficient number of observations to implement these methods. An alternative approach uses non-parametric estimators that do not assume an underlying frequency distribution and are relatively easy to compute. These estimators are implemented in DOTUR and MG-DOTUR.

**Table 1:** Tools used to describe and compare microbial communities.

**Tool**	**Application**	**Input**	**Reference**
DOTUR/MG-DOTUR	Assigns sequences to OTUs based on genetic distance between sequences and constructs rarefaction curves and collector's curves for richness and diversity estimators	Distance Matrix or BLAST Table	[30]
SONS	Generates collector's curves for estimates of the fraction and richness of OTUs shared between communities	OTU Designation	[56]
∫-LIBSHUFF/MG-LIBSHUFF	Tests whether the structures of two communities are the same, different, or subsets of one another using the Cramer-von Mises statistic	Distance Matrix or BLAST Table	[31, 32]
AMOVA/MG-AMOVA	Determines whether two or more communities differ significantly in genetic diversity using an analysis of variance-type formulation	Distance Matrix or BLAST Table	[33, 47, 48]

Based on the observed frequency distribution of peptide fragments in each OPF_0.30_, we applied the Chao1, ACE, and interpolated Jackknife richness estimators to predict the OPF_0.30 _richness. The predicted OPF richness was approximately three times greater than the OPF richness that was observed in the assembled *B. anthracis *genome (Table 1). When we mapped each OPF from the closed genome to the OPFs from the individual sequence reads we found that each OPF from the closed genome was linked to an average of 3.08 (s.d. = 2.75) OPFs from the sequence reads. Further inspection showed that the multiple OPFs from the sequence reads corresponded to different regions of long ORFs from the closed genome sequence. Similar results have been observed when attempting to estimate the number of expressed genes using expressed sequence tags [38].

**Figure 1:**
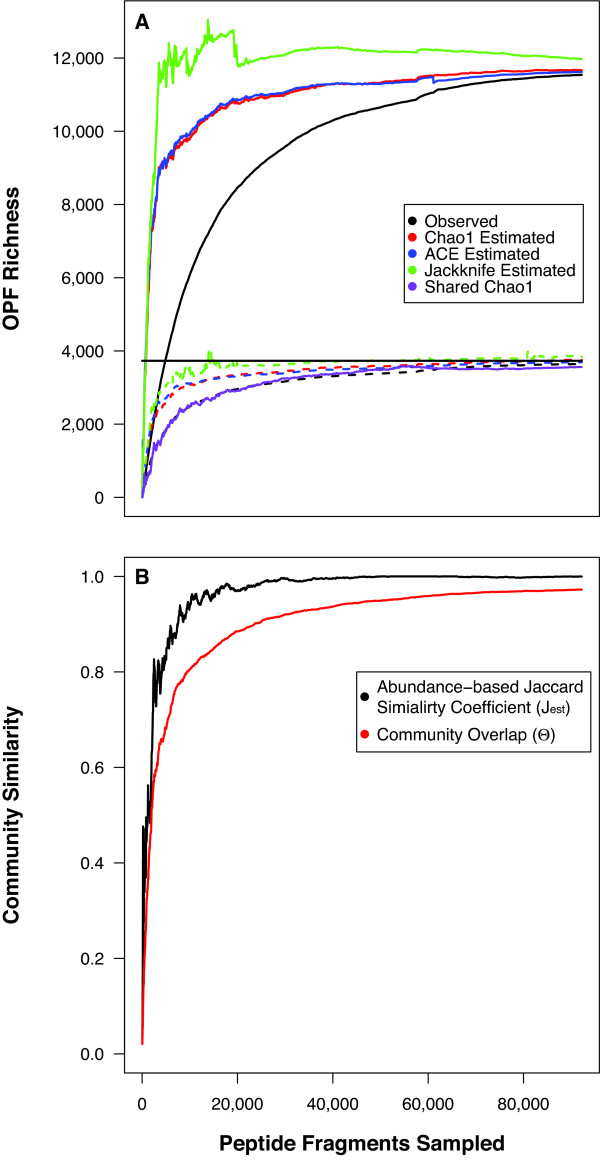
Analysis of the richness and community membership when peptide fragments identified in individual sequence reads were used to assemble the *Bacillus anthracis *str. Ames genome sequence. (A) The collector's curves for three non-parametric richness estimators and observed richness using individual sequence reads compared to the OPF richness of the assembled genome (horizontal black line). The solid lines represent the richness of non-merged OPFs and the dashed lines represent the richness of merged OPFs with a penalty of 0.15. (B) Collector's curves of parameters describing the similarity between two randomly selected subsets of peptide fragments.

To overcome this problem, we developed a method of merging OPFs from the sequence reads to obtain a more meaningful OPF distribution. For two OPFs to merge, we required that the carboxyl-terminus of at least one sequence in the first OPF overlap with the amino-terminus of at least one sequence in the second OPF by at least 5 amino acids. Furthermore, we incorporated a BSR penalty so that for two OPFs to merge the overlapping region had to have a BSR greater than the BSR currently being used to form clusters. We used penalties of 0.00, 0.05, 0.10, 0.15, and 0.20 (Table 1). We then applied this merging scheme to the OPFs from the sequence reads and calculated two types of error [38]. Type I errors corresponded to the fraction of OPFs from the closed genome that mapped to multiple OPFs from the sequence reads. Type II errors corresponded to the fraction of OPFs from the sequence reads that corresponded to different OPFs from the closed genome (Table 2). We found that as we increased the penalty, the Type I error decreased and the Type II error increased. Based on this analysis, we decided to implement a penalty of 0.15 because both types of error were 7.1 and 7.4%, respectively. When the resulting frequency distribution was used to calculate collector's curves using the observed and predicted richness, the curves converged towards the true OPF richness (Fig. 1A). This was used to further validate the choice of penalty. A limitation of this approach is that the resulting number of peptide fragments in a merged OPF is a product of the length of the complete ORF and the relative abundance of the ORF in the metagenome. Therefore, we will report OPF richness from merged analysis and annotations from both merged and non-merged analyses.

**Table 2:** Summary of errors and richness estimates when different criteria were used to merge OPFs. OPFs were merged when at least one peptide fragment in each OPF overlapped at least 5 aa and had a BSR value that was above the user specified level by the merge penalty. The type I error rate is the fraction of OPFs from the closed genome that correspond to multiple OPFs from the individual sequence reads. The type II error rate is the fraction of OPFs from the individual sequence reads that corresponded to more than one OPF from the closed genome sequence.

**Merge Penalty**	**Type I Error Rate**	**Type II Error Rate**	**Observed Richness**	**Richness Estimation (True Richness = 3,730)**		
				
				**Chao1**	**ACE**	**Jackknife**
Penalty = 0.00	0.063	0.129	2,927	3,038	2,976	3,137
Penalty = 0.05	0.067	0.100	3,223	3,332	3,271	3,413
Penalty = 0.10	0.071	0.074	3,462	3,574	3,510	3,653
Penalty = 0.15	0.080	0.056	3,642	3,757	3,691	3,839
Penalty = 0.20	0.091	0.046	3,810	3,925	3,858	4,004

No merge	0.719	0.003	11,538	11,668	11,616	11,968

### Comparing membership and structure using OPFs

Other tools have been developed to compare the membership (e.g. SONS) and structure (e.g. ∫-LIBSHUFF and AMOVA) of microbial communities using 16S rRNA gene sequences. Again, by analogy we were interested in using OPFs and BSRs to compare microbial communities using metagenomic sequences. SONS uses the output of DOTUR and MG-DOTUR to complete its analysis and required no further modification for use with metagenomic sequences. ∫-LIBSHUFF and AMOVA were modified to use the sparse matrix data representation used in MG-DOTUR. The resulting programs were designated MG-LIBSHUFF and MG-AMOVA. To test these programs, we randomly divided the 92,220 *B. anthracis *peptide fragments into two artificial communities that were each represented by 46,110 peptide fragments.

We first applied SONS to these two communities to compare the membership and structure of the artificial communities using OPFs. We calculated the shared OPF richness using the Chao non-parametric estimator of shared richness and obtained a value of 3,561 OPFs. Although this estimate of shared richness is lower than 95% confidence interval observed for the total collection of peptide fragments using the Chao1, ACE, or Jackknife estimators, the shard Chao estimator was still increasing with additional sampling (Fig. 1B). This indicates that if sequencing had continued the estimate of shared richness would have probably overlapped eventually. The abundance-based Jaccard (J_abund_) estimate of similarity was 1.00, which predicted that all of the peptide fragments belonged to shared OPFs_0.30_. Yue and Clayton's measure of community overlap, *θ*, was 0.97, which indicated that the distribution of peptide fragments among OPFs was the same in both artificial communities. These results indicate that SONS is amenable to analyzing OPFs to detect similarity between the memberships and structures of different communities.

An alternative approach to comparing community structures is to perform hypothesis tests. AMOVA uses an analysis of variance (ANOVA)-type framework to test the hypothesis that the difference in genetic diversity between two or more communities is not significantly different than the diversity within each community. We implemented this analysis in a program designated MG-AMOVA to perform a single-classification analysis. Our comparison of two randomly generated *B. anthracis *peptide fragment pseudo-communities revealed that the observed differences between the two pseudo-communities were not statistically significant (p > 0.05). Next we modified the program ∫-LIBSHUFF to create MG-LIBSHUFF to test the hypothesis that two communities have the same structures. As expected, the differences in structure between the two pseudo-communities were not statistically significant (P > 0.05). Each of these comparisons indicate that we can make statistical comparisons between the membership and structure of microbial communities using peptide fragments identified in single sequence reads from metagenomic data.

### Acid Mine Drainage

**Figure 2:**
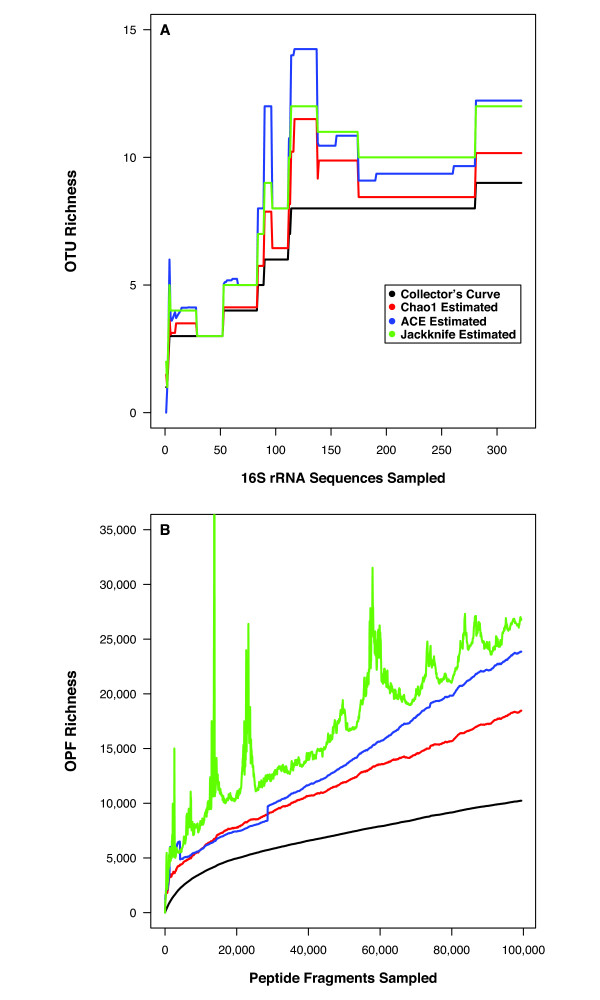
Collector's curves for the OTU (A) and OPF (B) richness observed and estimated using DNA extracted from an AMD biofilm community.

Tyson et al. [15] used metagenomic sequencing to analyze a biofilm growing in acid mine drainage (AMD) that had a pH below 1.0. They obtained 322 archaeal and bacterial 16S rRNA gene sequences and 103,462 random paired sequence reads, which represented 76.2 Gbp of DNA. We used DOTUR to assign 16S rRNA gene sequences to nine OTUs and predicted there were an additional three OTUs (95% confidence interval [95% CI] = 0 to 8) that were not observed (Fig. 2A). The most abundant OTU was similar to *Leptospirillum ferriphilum *(n = 247) 16S rRNA gene sequences.

Next, we used MG-DOTUR to assign 99,419 peptide fragments to 10,235 merged OPFs. The dominant merged OPF (n = 901 fragments) did not have a homolog in GenBank and the next most abundant merged OPFs_' _were most similar to a conserved hypothetical protein from *Leptospirillum *sp. Group II UBA (n = 773, EAY56482) and a transposase (n = 461, ZP_00669012). The dominant non-merged OPF did not have a homolog in GenBank (n = 114 fragments) and the next most abundant OPFs were most similar to an HNH nuclease (n = 96, ZP_01023224) and a mutator-type transposase (n = 88, ZP_00669012). The Chao1 richness estimator predicted that there were a minimum of 18,463 merged OPFs_0.30 _in the community (95% confidence interval [95% CI] = 17,794 to 19,191; Fig. 2B). Considering the lack of a known function for two of the most abundant OPFs in the AMD community, this analysis shows the importance of including such sequences in metagenomic sequence analyses and may indicate that subsequent analysis of this group of sequences would reveal important physiological information about the community.

### Soil

Tringe et al. [18] used Minnesotan farm soil to build libraries and sequence 1,633 bacterial 16S rRNA gene fragments and 149,085 random DNA fragments, representing 76 Gbp of DNA. We previously showed that the OTU richness was approximately 2,000 [39]. The three most abundant OTUs were representatives of the Chloroflexi.

**Figure 3:**
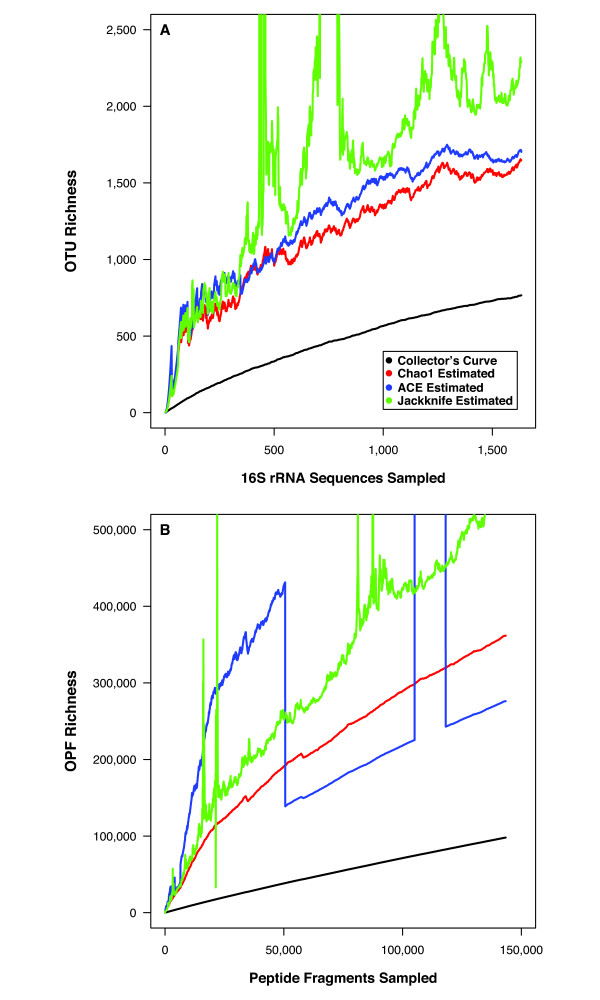
Collector's curves for the OTU (A) and OPF (B) richness observed and estimated using DNA extracted from an agricultural soil in Minnesota, USA.

Using MG-DOTUR to analyze the random metagenomic sequence reads, the 143,422 peptide fragments clustered into 98,066 merged OPFs. The members of the dominant merged OPF had similarity to a putative two-component response regulator (n = 688; NP_254170). The next most abundant merged OPFs had similarity to a histidine kinase (n = 566; YP_386369) and a serine/threonine protein kinase (n = 371; YP_825781). The three most abundant non-merged OPFs in the soil community had homology to a putative response regulator (n = 29, NP_520928), a PadR-like transcriptional regulator (n = 21, ZP_00524755), and a Cu^2+^-transporting ATPase (n = 20, ZP_01060472). Because of the considerable diversity in the soil sample, an insufficient number of peptide fragments were sampled to obtain a reliable OPF richness estimate; however, using the Chao1 richness estimator we predicted that the OPF richness was at least 361,546 (95% CI = 355,613 to 367,615; Fig. 3B). Although considerable additional sequencing effort is required to obtain a reliable estimate of OPF richness, it is interesting that in spite of the relatively large OTU and OPFs richness, it was possible to assign a large number of peptide fragments to the same OPF.

### Whalebone communities

Tringe et al. [18] compared three bacterial communities growing on the bones of two whales (AHAA and AHAI were from the same whale) at the bottom of the Pacific Ocean using 16S rRNA and metagenomic sequence analysis. Based on 16S rRNA sequence data, the three communities designated AGZO (n = 73), AHAA (n = 65), and AHAI (n = 68) had a Chao1-estimated OTU richness of at least 140 (95% CI = 67 to 366), 48 (95% CI = 29 to 121), and 19 (95% CI = 17 to 34). The most abundant OTU_0.03 _from each of the three communities affiliated with members of the *Arcobacter *sp. (n = 15), Bacteroidetes (n = 12), and Flavobacteriales (n = 19), respectively. We estimated that each of the three communities shared between 1 and 3 OTUs_0.03 _with any of the other communities. The lack of conservation of membership between the three communities resulted in low J_abund _coefficients (0.01 to 0.19), *θ *values (0.04 to 0.11), and statistically significant P values when comparing the communities using AMOVA and ∫-LIBSHUFF (all p < 0.001). Although the three communities each came from similar environments, the taxonomic membership and structure of the three communities were considerably different.

We applied the newly developed statistical tools to the metagenomic sequences of the three communities to assess their genetic and functional similarities. The three communities, AGZO, AHAA, and AHAI, yielded approximately 38,000 (25 Mbp), 38,000 (25 Mbp), and 40,000 (25 Mbp) sequence reads and 38,981, 36,165, and 33,199 peptide fragments, which were over 100 aa long, respectively. The dominant merged OPFs in each community were similar to a histidine kinase (AGZO, n = 386; YP_341128) and an ABC transporter (AHAA, n = 175 and AHAI, n = 166; ZP_01203057). The most abundant non-merged OPF found in each community was homologous to a conserved hypothetical protein (AGZO: n = 22, NP_442017), RecR (AHAA: n = 9, ZP_00952890), another conserved hypothetical protein (AHAI: n = 16, ZP_00949155), and a putative transposase (AHAI: n = 16, ZP_00903285). The Chao1 OPF richness estimates for each of the communities continued to increase with additional sampling, indicating that the communities had a minimum OPF richness of 69,541 (95% CI = 67,618 to 71,550), 77,923 (95% CI = 75,699 to 80,276), and 49,120 (95% CI = 47,767 to 50,539) for the AGZO, AHAA, and AHAI communities, respectively.

**Figure 4:**
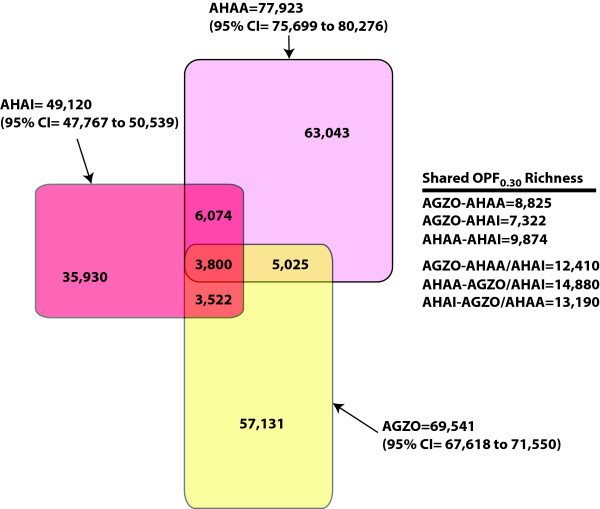
Venn diagram comparing the OPF membership found in three whalebone microbial communities (AGZO, n = 38,981 peptide fragments; AHAA, n = 36,165; and AHAI, n = 33,199). Below each community name is the Chao1 richness estimate and the 95% confidence interval for that community. We estimated the richness of the overlapping regions based on the pairwise S_A,B Chao _shared richness estimates between the three communities and by pooling two communities and estimating the shared fraction with the third community. These estimates are provided on the right side of the figure.

Although there was an insufficient number of peptide fragments to obtain a reliable estimate of the fraction of OPF membership that was shared between any two of the three communities, we estimated that they shared at least between 10 and 20% of their OPF membership (Fig. 4). The "core" whalebone OPF membership that was shared among the three whalebone communities had a richness of at least 3,800 OPFs (approximately 2.5% of the total richness); 1,678 of these were actually observed in the sequence collection. The most commonly shared OPFs among the three communities represented a variety of activities including metal transport, sensors, and housekeeping functions (Table 3).

**Table 3:** Summary of most abundant merged and non-merged OPFs from the three whalebone communities.

**Number of ORFs in OPF**			**Putative annotation**	**Representative GenBank Accession**
		
**AGZO**	**AHAA**	**AHAI**		
***Merged OPFs***				

386	32	26	Histidine kinase	YP_341128
229	175	166	ABC transporter	ZP_01203057
137	21	22	Aerotaxis sensor receptor	YP_339458
91	27	33	Sensory box protein	YP_341105
75	39	65	ATP-dependent RNA helicase protein	NP_518660
74	62	51	Translation elongation factor	ZP_01061839
56	85	104	Acyl-CoA dehydrogenase	ZP_01106089
52	68	66	Aldehyde dehydrogenase	YP_341708
49	65	93	Copper transport membrane protein	ZP_01060525
49	53	72	Acetyl-CoA acetyltransferase	ZP_01165108
44	45	66	Cation efflux protein	YP_678123

***Non-merged OPFs***				

8	5	3	Thioredoxin	ZP_01901399
7	3	5	Conserved hypothetical protein	ZP_01054178
6	5	3	GTP-binding protein LepA	YP_745328
6	3	9	DNA topoisomerase IV, subunit A	YP_756797
5	6	3	50S ribosomal protein, L20	ZP_01108363
5	6	3	50S ribosomal protein, L14	ZP_00952078
5	4	7	30S ribosomal protein, S11	ZP_01302802
4	8	5	Recombination protein, RecR	ZP_00948629
3	4	6	DNA helicase, RuvB	YP_357747

Comparison of the community structures using the peptide fragments using MG-LIBSHUFF (all p < 0.001) and MG-AMOVA (all p < 0.001) found that the structures of these three communities were significantly different. Using OPFs, *θ *varied between 0.39 and 0.55 indicating that there was some similarity in community structure. The ability to quantify and assess the differences in communities without exhaustive sampling of the three whalebone communities indicates the importance of applying statistical methods to metagenomic sequence data. Such analyses make comparative metagenomics amenable to ecologically-based hypothesis testing.

### Comparison of the three environments

To assess the relative similarity of OTU_0.03 _membership between environments, we used DOTUR to cluster the 2,161 16S rRNA gene fragments from the AMD (n = 322), soil (n = 1,633), and whalebone communities (n = 206). No OTUs were shared between any two of the three communities; however, additional sampling may have identified OTUs that were shared between environments.

**Figure 5:**
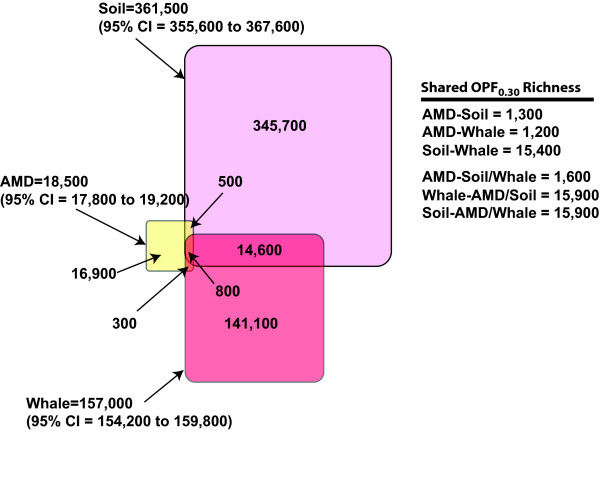
Venn diagram comparing the pooled OPF membership found in the AMD (n = 99,419 peptide fragments), soil (n = 143,422), and whalebone (n = 108,345) microbial communities.

We compared the relative similarity of OPF membership between environments by clustering the 351,186-peptide fragments from the AMD (n = 99,419), soil (n = 143,422), and whalebone communities (n = 108,345) using MG-DOTUR and then we estimated the membership and structure overlap among the three communities (Fig. 5). Measuring the overlap of OPFs measurement among the three communities resulted in the estimate that more than 800 OPFs were shared among the five communities; this represents less than 0.3% of the total OPF richness found in the five communities. Of this pool, 774 merged OPFs and were actually observed with functions including metal transport, housekeeping, and various dehydrogenase activities (Table 4). Applications of the statistical tools to these types of comparisons will enable researchers to investigate the problem of biogeography using genome-based methods.

**Table 4:** Summary of most abundant merged and non-merged OPFs from the AMD, soil, and whalebone communities.

**Number of ORFs in OPF**			**Putative annotation**	**Representative GenBank Accession**
		
**AMD**	**Soil**	**Whale**		
***Merged OPFs***				

562	350	451	Acetate CoA ligase	ZP_01856978
1628	1515	1240	Diguanylate cyclase signal protein	YP_001112705
796	1226	1086	ABC transporter	ZP_01060315
371	163	138	Resistance protein	ZP_01908921
216	121	152	Dehydrogenase	ZP_01454599
238	237	236	Cation transporting ATPase	ZP_01060472
237	170	269	Dehydrogenase	ZP_01105894
476	282	318	Translocation elongation factor	ZP_01594411
123	125	156	DNA helicase	ZP_01189997
169	184	289	Acyl CoA dehyodrogenase	ZP_01512967

***Non-merged OPFs***				

22	7	5	Urocanate hydratase	ZP_01709366
18	5	13	DNA gyrase, A subunit	ZP_01052578
17	9	12	Nucleoside-diphosphate kinase	ZP_01106957
11	13	14	Nitrogen regulatory protein PII	NP_767252
10	10	10	50S ribosomal protein, L19	YP_471434
9	12	9	GTP-binding protein LepA	ZP_01650030
7	11	14	50S ribosomal protein, L20	ZP_01108363
8	10	9	Excinuclease ATPase subunit	YP_861824
6	10	11	GMP synthase	ZP_01753395
5	13	10	30S ribosomal protein, S13	ZP_01885769

For comparison, we compared the complement of ORFs from the fully sequenced *Bacillus anthracis *str. 'Ames Ancestor' (GenBank accession AE017334), *Bacillus cereus *ATCC 10987 (AE017194), *Escherichia coli *K12 (U00096)*, Methanosarcina acetivorans *C2A (AE010299), *Methanosarcina barkeri *str. fusaro (CP000099) genomes. We used MG-DOTUR to assign ORFs to OPFs and then we used SONS to compare the OPF_0.30 _overlap between these genomes, which we selected for their phylogenetic similarity and breadth. As predicted based on current understanding of phylogenetics, the more closely related organisms had the greatest OPF_0.30 _overlap. The comparison between *B. anthracis *and *B. cereus *yielded J_clas _and *θ *values of 0.70 and 0.74, *E. coli *and *Y. pestis *yielded values of 0.43 and 0.20, and *M. acetivorans *and *M. barkeri *yielded values of 0.54 and 0.43. All of the other pairwise comparisons yielded values below 0.08 for both parameters. This analysis suggests that the comparisons between the OPFs_0.30 _identified in the metagenomic sequences represent the level of differences expected between phylogenetically disparate groups of bacteria. Furthermore, analyses using completed genome sequences may enable investigators to define the size and boundaries of so-called "pan-genomes."

## Conclusion

We present a statistical toolbox to estimate the functional richness and overlap among communities based on peptide fragments deduced from DNA sequence data. These statistical approaches are necessary, in part, because the immense genomic diversity contained in most communities precludes the formation of contigs. There is also considerable question regarding the robustness of sequence assembly [40]. Although understanding these complex communities is tantalizing, it may prove useful to identify more communities similar to the AMD and whalebone communities that have a relatively low diversity to develop and test tools that can then be applied to soil. As sequencing technologies improve, the feasibility of obtaining nearly complete sequence coverage of the more diverse communities will improve. The rapid advances in sequencing short DNA fragments (approximately 100 bp long) in a highly parallelized manner [41] presents many new opportunities, but the method may not be amenable to metagenomic sequencing because the short sequence reads produce peptide fragments less than 100 aa long, which could make a meaningful ORF identification and analysis of functional diversity difficult.

Innovative methods have been developed to compare collections of 16S rRNA sequences, and analogous new methods are needed for comparing metagenomic sequences. For example, improving our ability to estimate and interpret the biological meaning of OPF richness will be helpful for describing the relative functional capacity of a community. Our analysis does not address the possibility that distant OPFs might serve the same biological function and that members of the same OPF might have different functions. Therefore, further work is needed to unify studies of functionally active clones into a statistical framework. For example, comparing the collection of genes conferring antibiotic resistance found in multiple environments would enable us to understand better the diversity of these genes as well as their biogeography.

Our analysis moves beyond previous attempts to compare microbial communities at the genomic level by not being dependent upon reference databases and introducing statistical rigor to the description and comparison of microbial communities. For example, previous analyses formed clusters based on similarity to reference databases and excluded those peptide fragments with no significant matches, which limited the scope of the analysis. Here, we formed OPFs using the observed data, in essence allowing the data to "speak for themselves", which allowed for a comprehensive comparison of the data. Previous analyses also based the level of similarity between communities on the observed peptide fragments as though they represented a statistical population. Here, we treated the data as a statistical sample and employed statistical tools to estimate the level of similarity between community membership and structure. These tools enable a quantitative, comprehensive, and statistically robust analysis of microbial communities at the genomic level.

Shotgun sequencing of metagenomic communities is becoming increasingly popular and routine. The results of these efforts will provide more insight if they are wrapped in robust ecological and statistical frameworks. Tools are needed to advance data analysis beyond the frequency of different COGs or KEGG categories that are found within a community. This study is a step in building such a framework to compare microbial communities functionally at the genomic level. In addition to estimating community relatedness based on metagenomic data, our approach accounts for present but unsampled peptide fragments, is independent of a subjective annotation process, and includes peptide fragments with no known function.

## Methods

### Genome sequence data

We obtained the 101,379 sequence reads used to assemble the *Bacillus anthracis *str. Ames whole genome sequence from GenBank (NC_003997). Each sequence read was evaluated by fastgenesb at the Joint Genome Institute using the same parameters used to predict the identity of peptide fragments in two previous metagenomic sequencing studies [15,18]. We also obtained the complete complement of 4,514 ORFs from the finished genome that were longer than 100 aa. All of the predicted peptide fragments from the published metagenomic sequencing projects using an acid mine drainage biofilm [15], whalebone [18], and soil [18] were obtained from the Joint Genome Institute. Only those ORFs and peptide fragments longer than 100 aa were considered in our analyses.

### Modified toolbox

DOTUR is a freely available computer program that uses a distance matrix to assign sequences to operational taxonomic units (OTUs) using either the nearest, average, or furthest neighbor clustering algorithms for all possible distances and then constructs rarefaction and collector's curves for a variety of ecological parameters [30]. These curves can be used to compare the relative richness, the number of different OTUs in a community, of two samples and to estimate the overall richness within a sample. Similarly, MG-DOTUR clusters sequences into OPFs using a BLAST table as the input. ORFs are assigned to OPFs using the furthest neighbor clustering algorithm [42], which requires that all sequences in the OPF have a pairwise BSR value greater than a specified value. Because BSRs are not necessarily symmetric (i.e. BSR_ij _≠ BSR_ji_), they were forced to be symmetric by using the smaller of the two values. Once MG-DOTUR assigns sequences to OPFs, rarefaction curves of the number of OPFs observed on average as a function of ORFs sampled and collector's curves of the Chao1 [43], ACE [44], and the interpolated Jackknife [45] richness estimates as a function of ORFs sample are calculated at multiple BSR values predefined by the user. MG-DOTUR uses a switch to calculate the ACE estimator. If the coefficient of variation (*γ*) is greater than 0.8, then the ACE-1 estimator is calculated, otherwise the simple ACE estimator is used. This follows recommendations made by Anne Chao for use of the program SPADE [46]. This study reports results obtained by defining an OPF a group of sequences with a BSR value greater than 0.30 [34,35] and OTU as a group of sequences that are all more than 97% identical to each other [30].

∫-LIBSHUFF [31] is a modified version of the program LIBSHUFF [32] that makes use of the integral form of the Cramér-von Mises statistic to determine whether two communities are either samples of the same statistical population, sub-samples of each other, or were drawn from different statistical populations. As employed in ∫-LIBSHUFF, the Cramér-von Mises statistic is a function of the coverage of one sequence collection onto itself (i.e. homologous coverage, C_X_) compared to its coverage onto another collection (i.e. heterologous coverage, C_XY_). Coverage is the fraction of sequences that have another sequence within a given distance of them. Application of the LIBSHUFF-style analysis requires converting BSR values into distances by subtracting the BSR value from one and setting the limits of integration from zero to 0.70. MG-LIBSHUFF calculates the ΔC_XY _statistic and evaluates its significance using a Monte Carlo testing procedure as described elsewhere [31,32].

$$\Delta C_{XY}=\int_{0}^{1-BSR_{\min}}{\left(C_{X}(D)-C_{XY}(D)\right)}^{2}dD$$

where,

D = the distance (1-BSR) that is used to determine the level of coverage.

C_X_(D) and C_XY_(D) = measures of homologous and heterologous library coverage.

BSR_min _= the smallest meaningful BSR value; for this analysis set at 0.30

Population biologists have developed an analysis of variance (ANOVA)-style of analysis, which tests whether a collection of communities have similar genetic diversities using mitochondrial DNA sequences and other genetic markers. This method has been designated as either the analysis of molecular variance (AMOVA) [47] or non-parametric multivariate analysis of variance (MANOVA) [48]. This analysis has been applied for comparing bacterial communities using 16S rRNA sequences [33]. The general method is based on partitioning the sum of the squared elements in a distance matrix similar to what is done in an ANOVA. As applied in MG-AMOVA, we implement a single-classification ANOVA design to determine whether the average genetic average genetic difference between the three whalebone communities was significantly greater than the difference within a community. The total sum-squared error (SS_T_) and within community sum-squared error (SS_W_) is calculated by

$$\begin{array}{l} SS_{T}=\frac{1}{N}\sum_{i=1}^{N-1}\sum_{j=i+1}^{N}{\left(1-BSR_{ij}\right)}^{2}\\ SS_{W}=\frac{1}{N}\sum_{i=1}^{N-1}\sum_{j=i+1}^{N}{\left(1-BSR_{ij}\right)}^{2}\varepsilon_{ij} \end{array}$$

where,

BSR_ij _= the BSR value between the i^th ^and the jth peptide fragments.

*ε*_ij _= 1 if i and j are in the same community, otherwise it is 0.

N = total number of peptide fragments

The sum-squared error among communities (SSA) can be calculated as SS_A _= SS_T_-SS_W_. Significance was determined by randomizing the assignment of sequences to the sequence collections and recalculating the statistic and determining the proportion of randomizations resulting in an equal or smaller SS_W _value than that observed from the randomized distribution [48].

### OPF-based comparisons of community membership and structure

Using the frequency that each OPF was observed in multiple communities, it has been possible to estimate the number of OPFs that are shared between communities as well as describe the overlap between community structures. Analogous to the Chao1 non-parametric richness estimator [43], Chao et al. [49] derived a non-parametric estimator of the richness shared between two communities:

$$S_{A,B\;Chao}=S_{12}+f_{11}\frac{f_{1+}f_{+1}}{4f_{2+}f_{+2}}+\frac{f_{1+}^{2}}{2f_{2+}}+\frac{f_{+1}^{2}}{2f_{+2}}$$

where,

S_12 _= number of shared OPFs in A and B

f_11 _= number of shared OPFs with one observed individual in A and B

f_1+_, f_2+ _= number of shared OPFs with one or two individuals observed in A

f_+1_, f_+2 _= number of shared OPFs with one or two individuals observed in B

By a similar approach the fraction of individuals or peptide fragments that belong to a shared OPF can be estimated [50,51]:

$$\begin{array}{l} U_{est}=\sum_{i=1}^{S_{12}}\frac{X_{i}}{n_{total}}+\frac{m_{total}-1}{m_{total}}\frac{f_{+1}}{2f_{+2}}\sum_{i=1}^{S_{12}}\frac{X_{i}}{n_{total}}I\left(Y_{i}=1\right)\\ V_{est}=\sum_{i=1}^{S_{12}}\frac{Y_{i}}{m_{total}}+\frac{n_{total}-1}{n_{total}}\frac{f_{1+}}{2f_{2+}}\sum_{i=1}^{S_{12}}\frac{Y_{i}}{m_{total}}I\left(X_{i}=1\right) \end{array}$$

where,

U_est_, V_est _= fraction of sequences from A and B that belong to a shared OTU

X_i_, Y_i _= abundance of the i^th ^shared OTU in A and B

n_total_, m_total _= total number of sequences sampled in A and B

I(·) = if the argument, ·, is true then I(·) is 1; otherwise it is 0.

U_est _and V_est _can then be used to estimate an abundance-based Jaccard similarity coefficient:

$$J_{abund}=\frac{U_{est}V_{est}}{U_{est}+V_{est}-U_{est}V_{est}}$$

To incorporate into the measure of community similarity the proportion of peptide fragments in each OPF, Yue and Clayton [52] developed the parameter *θ*:

$$\theta =\frac{\sum_{i=1}^{S_{12}}\frac{X_{i}}{n_{total}}\frac{Y_{i}}{m_{total}}}{\sum_{i=1}^{S_{1}}{\left(\frac{X_{i}}{n_{total}}\right)}^{2}+\sum_{i=1}^{S_{2}}{\left(\frac{Y_{i}}{m_{total}}\right)}^{2}-\sum_{i=1}^{S_{12}}\frac{X_{i}}{n_{total}}\frac{Y_{i}}{m_{total}}}$$

where,

S_1 _and S_2 _= observed number of OPFs in each community.

### 16S rRNA sequence analysis

The three metagenomic sequencing projects were selected because they were accompanied by parallel 16S rRNA sequence collections. We obtained the sequences from the original authors and aligned the sequences using the greengenes website [53]. Aligned sequences were imported to ARB [54] and overlapping sequences were used to construct distance matrices with a Jukes-Cantor correction for multiple substitutions. Distance matrices were analyzed using DOTUR [30], ∫-LIBSHUFF [31], and MG-AMOVA as described above.

## Availability of data and software

MG-DOTUR, MG-LIBSHUFF, MG-AMOVA and all sequence and analysis files are available from the authors' website [55].

## Authors' contributions

PDS designed the study, developed the methods and software, analyzed results, and wrote the manuscript. JH analyzed results and wrote the manuscript. All authors read and approved the final manuscript.
